# Treatment with 5-Aza-2'-Deoxycytidine Induces Expression of NY-ESO-1 and Facilitates Cytotoxic T Lymphocyte-Mediated Tumor Cell Killing

**DOI:** 10.1371/journal.pone.0139221

**Published:** 2015-10-08

**Authors:** Agnes S. Klar, Jakka Gopinadh, Sascha Kleber, Andreas Wadle, Christoph Renner

**Affiliations:** 1 Tissue Biology Research Unit, Department of Surgery, University Children’s Hospital Zurich, Zurich, Switzerland; 2 Children’s Research Center, University Children’s Hospital Zurich, Zurich, Switzerland; 3 Department of Oncology, University Hospital Zurich, Zurich, Switzerland; 4 Department of Oncology, University Hospital Basel, Basel, Switzerland; University of Palermo, ITALY

## Abstract

**Background:**

NY-ESO-1 belongs to the cancer/testis antigen (CTA) family and represents an attractive target for cancer immunotherapy. Its expression is induced in a variety of solid tumors via DNA demethylation of the promoter of CpG islands. However, NY-ESO-1 expression is usually very low or absent in some tumors such as breast cancer or multiple myeloma. Therefore, we established an optimized *in vitro* treatment protocol for up-regulation of NY-ESO-1 expression by tumor cells using the hypomethylating agent 5-aza-2'-deoxycytidine (DAC).

**Methodology/Principal Findings:**

We demonstrated *de novo* induction of NY-ESO-1 in MCF7 breast cancer cells and significantly increased expression in U266 multiple myeloma cells. This effect was time- and dose-dependent with the highest expression of NY-ESO-1 mRNA achieved by the incubation of 10 μM DAC for 72 hours. NY-ESO-1 activation was also confirmed at the protein level as shown by Western blot, flow cytometry, and immunofluorescence staining. The detection and quantification of single NY-ESO-1 peptides presented at the tumor cell surface in the context of HLA-A*0201 molecules revealed an increase of 100% and 50% for MCF7 and U266 cells, respectively. Moreover, the enhanced expression of NY-ESO-1 derived peptides at the cell surface was accompanied by an increased specific lysis of MCF7 and U266 cells by HLA-A*0201/NY-ESO-1(_157–165_) peptide specific chimeric antigen receptor (CAR) CD8^+^ T cells. In addition, the killing activity of CAR T cells correlated with the secretion of higher IFN-gamma levels.

**Conclusions/Significance:**

These results indicate that NY-ESO-1 directed immunotherapy with specific CAR T cells might benefit from concomitant DAC treatment.

## Introduction

Cancer immunotherapy has emerged as an alternative or adjuvant/supplement approach for cancer treatment [1,2]. Due to its weak side effects and favorable applicability, immunotherapy holds promise in stimulating patient’s own immune response to specifically target tumor cells. In this regard, tumor antigens called cancer/testis antigens (CTAs) represent promising therapeutic targets for cancer vaccination [3,4,5]. They are expressed only in immune privileged germ cells (lacking MHC class I molecules) and are also frequently expressed in various types of human tumors [3,4,5]. In particular, NY-ESO-1 is the most spontaneously immunogenic CTA described so far [5,6].

It has been shown that expression of NY-ESO-1 is frequently reactivated in tumor cells and elicits spontaneous humoral and cellular immune responses in some cancer patients [7]. Unfortunately, NY-ESO-1 expression is often heterogeneous within a tumor and sometimes too weak to induce a strong immune recognition [8,9]. Relatively few studies have focused on the expression pattern of NY-ESO-1 antigen in breast cancer and its protein expression was reported to be very low [10,11]. Specific antibodies against NY-ESO-1 were found only in 4% of the breast cancer patients [10]. To overcome this limitation, we aimed to enhance NY-ESO-1 expression.

Treatment of tumor cells with demethylating agents such as 5-aza-2’-deoxycytidine (DAC) was shown to increase or even induce *de novo* expression of several CTAs in various cancer types *in vitro* and *in vivo* [12,13,14,15,16]. This induction or up-regulation of CTAs expression is associated with promoter region hypomethylation [17]. The increased expression of NY-ESO-1 following the treatment with DAC has been demonstrated to trigger a functional recognition by antigen-specific cytotoxic T-lymphocytes (CTLs) [16,18]. In addition, promoter demethylation has also been shown to up-regulate the expression of major histocompatibility complex (MHC) class I molecules, which leads to an increased recognition by specific CTLs [19,20,21,22].

To date, very little is known, whether the observed increase in cytotoxicity is solely due to the increase of the total number of HLA molecules at the cell surface or due to a specific increase of the proportion of the respective peptide presented in the HLA groove. Our group has previously developed high-affinity peptide/MHC-specific antibody fragments recognizing the NY-ESO-1_157−165_ peptide in the context of the HLA-A*0201 complex [23]. This allows for a detailed monitoring of the presented peptide level at the cell surface. Furthermore, when grafted as a chimeric antigen receptor (CAR/T cell), the antibodies conferred specific killing of HLA-A*0201/NY-ESO-1_157–165_ expressing target cells [24,25,26].

Here, we provide a detailed analysis of the effects of 5-aza-2’-deoxycytidine (DAC)-treatment on NY-ESO-1 mRNA, protein, and presented peptide levels of tumor cells. We quantified the displayed NY-ESO-1_157−165_ peptides in the context of HLA-A*0201 complex before and after treatment and show immunotherapeutic implications for chimeric antigen receptor (CAR) grafted T cells.

## Materials and Methods

### Cells

The human melanoma cell lines: SK-MEL-23 [27] (NY-ESO-1^-^, HLA-A*0201^+^), SK-MEL-37 [28] (NY-ESO-1^+^, HLA-A*0201^+^), human breast cancer cell lines: ALAB [29] (NY-ESO-1^+^, HLA-A*0201^+^), MCF7 [30] (NY-ESO-1^-^, HLA-A*0201^+^), 734B [31] (NY-ESO-1^-^, HLA-A*0201^+^), multiple myeloma cell lines: ARK [32] (NY-ESO-1^+^, HLA-A*0201^-^), U266 [32] (NY-ESO-1^+^, HLA-A*0201^+^), and T2-1B cells (HLA-A*0201-positive T2 cells stably transfected with the HLAA*02:01-restricted NY-ESO-1 peptide 157–165) [23] were used (all kindly provided by Prof. Dr. van den Broek, Institute of Experimental Immunology, University of Zurich, Switzerland). Tumor cell lines were cultivated in standard R10 media (RPMI1640) GlutaMax (Invitrogen, Karlsruhe, Germany) at 37°C in a humidified atmosphere of 5% CO2 in air. The medium was supplemented with 10% fetal bovine serum (FBS) (v/v), 50 U/ml penicillin, and 50 μg/ml streptomycin; all obtained from Invitrogen (Karlsruhe, Germany).

### Reagents, anti-idiotypes, and antibodies

A vial containing 5 mg lyophilized powder of 5-aza-2'-deoxycytidine was obtained from Sigma-Aldrich Chemie GmbH (Buchs, Switzerland). The vial was reconstituted with sterile water to obtain a 1 mM solution and was stocked at 4°C. Antibodies for flow cytometry were purchased from Invitrogen (Karlsruhe, Germany) (anti-human NY-ESO-1, clone: E978), BD Biosciences (Allschwill, Switzerland) (anti-human HLA-A2-PE, clone: BB7.2, anti-mouse κ-isotype control IgG2a-PE), Sigma-Aldrich Chemie GmbH (Buchs, Switzerland) (anti-human α-Tubulin, clone: B-5-1-2, anti-mouse IgG (H+L)-HRP). QuantiBrite PE beads were purchased from BD Biosciences (Allschwill, Switzerland) and Streptavidin, R-phycoerythrin conjugated (SAPE) from Invitrogen (Karlsruhe, Germany). Immunofluorescence staining was performed using the T1 antibody recognizing the HLA-A*0201/NY-ESO-1_157–165_ peptide complex [24], the control antibody (AffiniPure anti-mouse IgG, Fc specific), and secondary antibody (AffiniPure F(ab)_2_ Frag anti-human IgG-PE) were purchased from Jackson ImmunoResearch (Suffolk, UK).

### *In vitro* treatment of cultured cells with DAC

Treatment with 5-aza-2'-deoxycytidine (DAC) was performed as described in a previous study [12]. Cells were seeded at a density of 1.0 x 10^6^ cells in T25 flasks and placed at 37°C overnight in a 5% CO2 incubator. The next day, the cell culture medium was replaced with fresh medium containing 0, 1, 7, 10, and 15 μM DAC. The medium was changed 1, 2, 3, and 4 times daily for 1–3 days. After the treatment, the medium was replaced with fresh medium without DAC and the cells were cultured for additional 48 h.

### Generation of anti HLA-A*02:01/NY-ESO-1_157–165_ CAR redirected T cells

NY-ESO-1_157–165_ peptide and CEA specific CAR redirected CD8^+^ T cells were generated as described previously [25,26]. Briefly, peripheral blood mononuclear cells (PBMCs) were isolated by Ficoll gradient from blood donated by healthy volunteers. Informed written consent was obtained from all blood donors at the Zurich Blood Doning Center (Blutspende Zürich, Rütistrasse 19, 8952 Schlieren, Switzerland, www.zhbsd.ch) according to the guidelines of the Ethics Committee of the Government of Zurich, Kantonale Ethikkommission Zürich (KEK), Sonneggstrasse 12, CH-8091 Zürich, Switzerland. CD8^+^ T cell isolation kit (Miltenyi Biotech, Germany) was used to isolate CD8^+^ T cells by negative magnetic bead sorting according to the manufacturer’s instructions. CD8^+^ T cells were retrovirally transduced with recombinant receptors by co-culturing the polyclonally activated CD8^+^ T cells with transiently transfected 293T cells as described [25]. After 24 h of co-cultivation, expression of CAR was confirmed by flow cytometry using phycoerythrin (PE)-labeled anti-human IgG1, HLA-A*0201/NY-ESO-1_157−165_ PE-tetramer or fluorescein isothiocyanate (FITC)-anti-human CD8 (Biolegend, San Diego, CA, USA) (S4 Fig).

### Activation of NY-ESO-1_157−165_ specific CAR redirected T cells

NY-ESO-1_157−165_ and CEA specific CAR redirected CD8^+^ T cells were co-cultured in 96-well round bottom microtiter plates at different numbers of effector (ranging from 5000–20.000 CAR-positive T cells per well) with 10.000 of target cells (untreated or DAC-treated MCF7, U266, and T2-1B cells) in 200 μl of R10 medium. After 24 h, XTT reagent (Cell Proliferation Kit II, Roche Diagnostics, Rotkreuz, Switzerland) was added to the cells and incubated at 37°C for 30–90 min. Reduction of XTT to formazan by viable cells was colorimetrically monitored [26]. Cell viability was calculated as follows: cell viability = [OD (experimental wells—corresponding number of effector cells / OD (cancer cells without effector cells—medium] x 100. Antigen specific IFN-gamma secretion by NY-ESO-1_157−165_ CAR T cells was measured in the culture supernatant by using an IFN-gamma ELISA kit (BD OptEIA™, San Diego, CA, USA) according to the manufacturer's instructions.

### Flow cytometry

Flow cytometry was carried out as previously described [24]. Briefly, cells were washed in FACS buffer (PBS, 2% heat-inactivated FCS, 5mM EDTA, 0.01% sodium azide), stained with directly labeled or with peptide-MHC class I mono-/tetramers for 30 min at room temperature (RT). Thereafter, cells were washed, resuspended in FACS buffer, and analyzed by flow cytometry using FACSCan equipment (BD Biosciences, San Diego, CA). Data were analyzed using WinMDI software. Geometric mean was chosen as mean fluorescence intensity (MFI). In some studies, the MFI of cells was compared with the MFI from a standard curve of PE-coupled calibration of QuantiBRITE PE beads carrying known quantities of PE molecules per bead and an estimated amount of PE molecules per stained cell was determined (S2 Fig). Anti-HLA-A*0201/NY-ESO-1_157–165_ Fab-monomers were used for quantifications of NY-ESO-1 peptides on cells, whereas for all other applications Fab-tetramers were used.

### Immunofluorescence staining

Cells were cultured and stained directly on 8-well chambered Ibidi coverslips (Martinsried, München, Deutschland) with directly labeled antibodies or anti-HLA-A*02:01/NY-ESO-1_157–165_ mono-/tetramers, and incubated for 30 min at RT. Cells growing in suspension (U266) were stained first and then spun down. After washing, cells were resuspended in PBS and analyzed using a Zeiss 200M microscope (Carl Zeiss AG, Munich, Germany).

### Western blotting

Cancer cells treated with DAC (10 μm, 4x a day, for 3 days) and untreated cells were lysed in a RIPA buffer containing protease inhibitors reagent (Bio-Rad, Cressier, Switzerland). Protein samples were denatured at 95°C for 5 min and subsequently applied to each well and electrophoresed on a 12% polyacrylamide gel. After transferring the proteins to a nitrocellulose membrane (Whatman, 0.2 μm) (Sigma-Aldrich Chemie GmbH (Buchs, Switzerland), it was blocked for 1 h at RT with 2% low-fat skim milk, and incubated with a monoclonal antibody (mAb) against NY-ESO-1 (clone: E978; 1:1000) and anti-human α-Tubulin (clone: B-5-1-2; 1:1000) overnight at 4°C. The membrane was then washed three times at 10 min intervals with buffer (PBS plus 0.3% Tween 20) and incubated with an horseradish peroxidase (HRP)-labeled secondary anti-mouse Ab (Bio-Rad, Cressier, Switzerland) for 1 h at RT. The washing steps were repeated, and the results were visualized using an enhanced chemiluminescence technique.

### RNA extraction and preparation of cDNA

Total RNA was extracted using the RNeasy Mini Kit (Qiagen, Valencia, CA) and was subsequently digested with DNAse I (Invitrogen, Karlsruhe, Germany). The concentration and purity was evaluated using the NanoDrop ND-1000 spectrophotometer (NanoDrop Technologies, Wilmington, DE). RNA was reverse transcribed using the SuperScript III Reverse Transcriptase Kit (Invitrogen, Karlsruhe, Germany). All kits were used according to the manufacturer’s instructions. The cDNA was either used immediately for RT-qPCR or stored at -20°C until use.

### Quantitative RT-PCR for NY-ESO-1 expression

After synthesis of the cDNA, quantitative RT-PCR was performed on the ABIPRISM^®^ 7000 thermal cycler (Applied Biosystems, Foster City, Ca). The expression of NY-ESO-1 was analyzed using TaqMan „assay on demand”primers and TaqMan 1x universal master mix (Applied Biosystems). 18s rRNA was used as a housekeeping gene. The reaction mixture (10 μL) consisted of 3.5 μl water, 1 μl cDNA, 0.5 μl primers (NY-ESO-1 or 18s rRNA) and 5 μl TaqMan universal master mix. Each sample was tested in three independent experiments and loaded in triplicates for each of the qRT-PCR experiments. Murine NY-ESO-1 negative cancer cell line (EL-4) and water controls were used as negative controls. The following cycle conditions were used: 2 min 50°C, 10 min 95°C, 45 cycles of 15 s at 95°C, 1 min 60°C.

### Statistical analysis

All results are reported as mean ± standard deviation (SD). Statistical analysis was performed with GraphPad Prism 4.0 (Graph Pad software, La Jolla, CA, USA). Comparison between two groups was performed using the unpaired Student’s t test. Results were considered significant according to GraphPad Prism:

* indicates p-value 0.01 to 0.05 (significant)

** indicates p-value 0.001 to 0.01 (very significant)

*** indicates p<0.001 (extremely significant)

ns = not significant (p>0.05)

## Results

### Effects of DAC treatment on HLA-A2/NY-ESO-1p_157-165_ peptide presentation on cancer cells

The NY-ESO-1_157–165_ and HLA-A*0201 (HLA-A2) status of different human cancer cell lines: melanoma cell lines: SK-MEL-23 (NY-ESO-1^-^, HLA-A*0201^+^), SK-MEL-37 (NY-ESO-1^+^, HLA-A*0201^+^), breast cancer cell lines: ALAB (NY-ESO-1^+^, HLA-A*0201^+^), MCF7 (NY-ESO-1^-^, HLA-A*0201^+^), 734B (NY-ESO-1^-^, HLA-A*0201^+^), and multiple myeloma cell lines: ARK (NY-ESO-1^+^, HLA-A*0201^-^), U266 (NY-ESO-1^+^, HLA-A*0201^+^) was analyzed by flow cytometry and summarized in S1A Fig. The susceptibility of the indicated cancer cell lines to DAC treatment was evaluated in S1B Fig. Accordingly, only some of the tested cancer cell lines—MCF7 and U266—responded to DAC treatment showing a *de novo* or up-regulated NY-ESO-1 expression.

**Fig 1:**
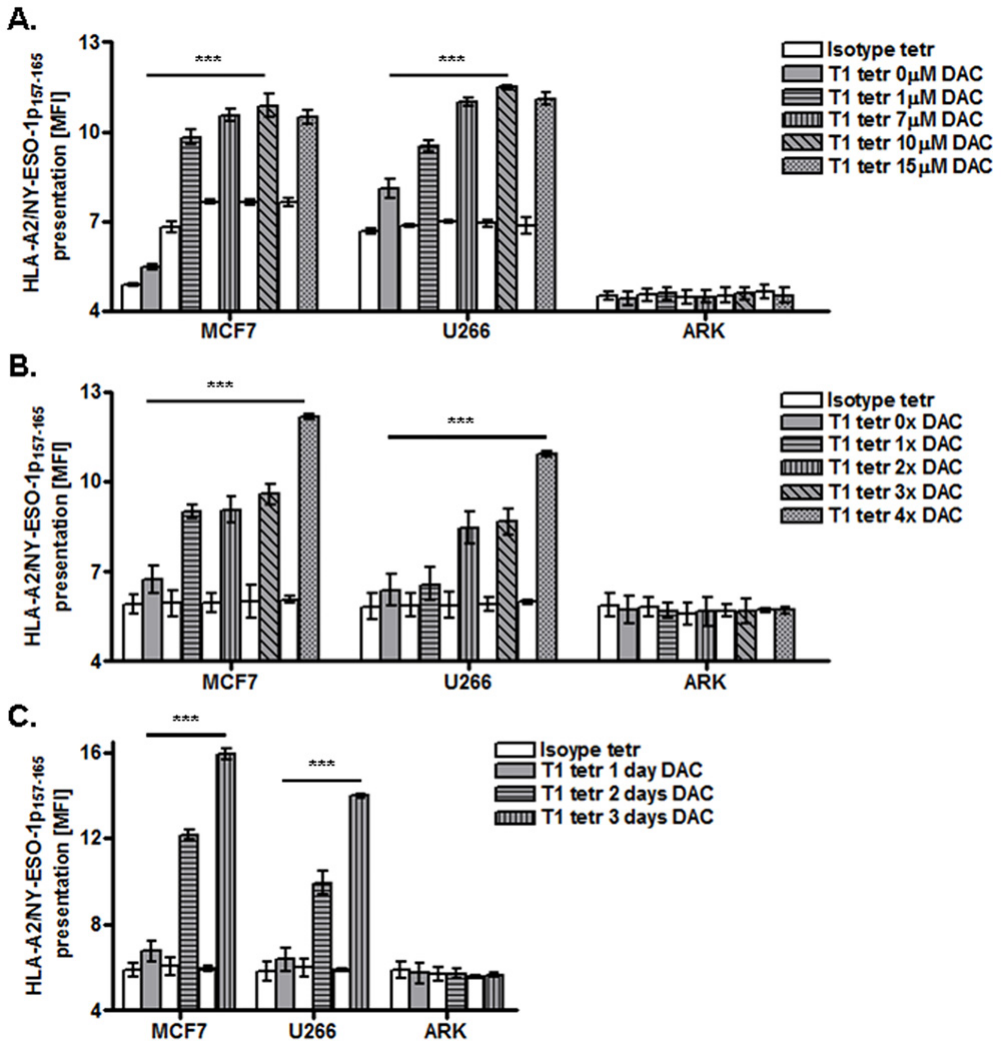
Optimization of DAC treatment for MCF7, U266, and ARK tumor cell lines. A. Variation of DAC concentration in cell culture medium as indicated (0–15 μM); white bars: isotype control, grey bars: detection with HLA-A2/NY-ESO-1p_157-165_ specific Fab-tetramers. B. Variation of DAC-treatment intensity (0–4 times per day). C. Variation of DAC-treatment duration (1–3 days). All data are representative of at least three independent experiments performed in triplicate.

To obtain optimal HLA-A2/NY-ESO-1p_**157-165**_ peptide presentation at the tumor cell surface, we optimized following parameters of the DAC treatment: DAC concentration, frequency of DAC applications, and DAC treatment duration (n = 3) (Fig 1). First, we assessed the effect of different DAC concentrations (1 μM, 5 μM, 7 μM, 10 μM, and 15 μM) on cultured cancer cells (Fig 1A). The DAC concentration ranging from 1 to 7 μM caused a slight but consistent increase in antigen presentation on MCF7 and U266 cells. However DAC doses of 15 μM reduced this expression. ARK cells served as a negative control due to the lack of HLA-A2 expression. The analysis of various frequencies of DAC treatment showed clearly that the DAC application four times daily increased the strongest antigen presentation on both MCF7 and U266 cells (Fig 1B). In comparison, in breast cancer cells both single, double, and triple DAC application per day induced only a slight up-regulation of the peptide presentation by about 23%. In NY-ESO-1 expressing U266 multiple myeloma cells, however, only a single DAC application per day did not induce higher antigen expression than the basal level before treatment (Fig 1B). Two and three times DAC application daily increased somewhat the antigen presentation, but weaker than four times per day treatment (Fig 1B).

**Fig 2:**
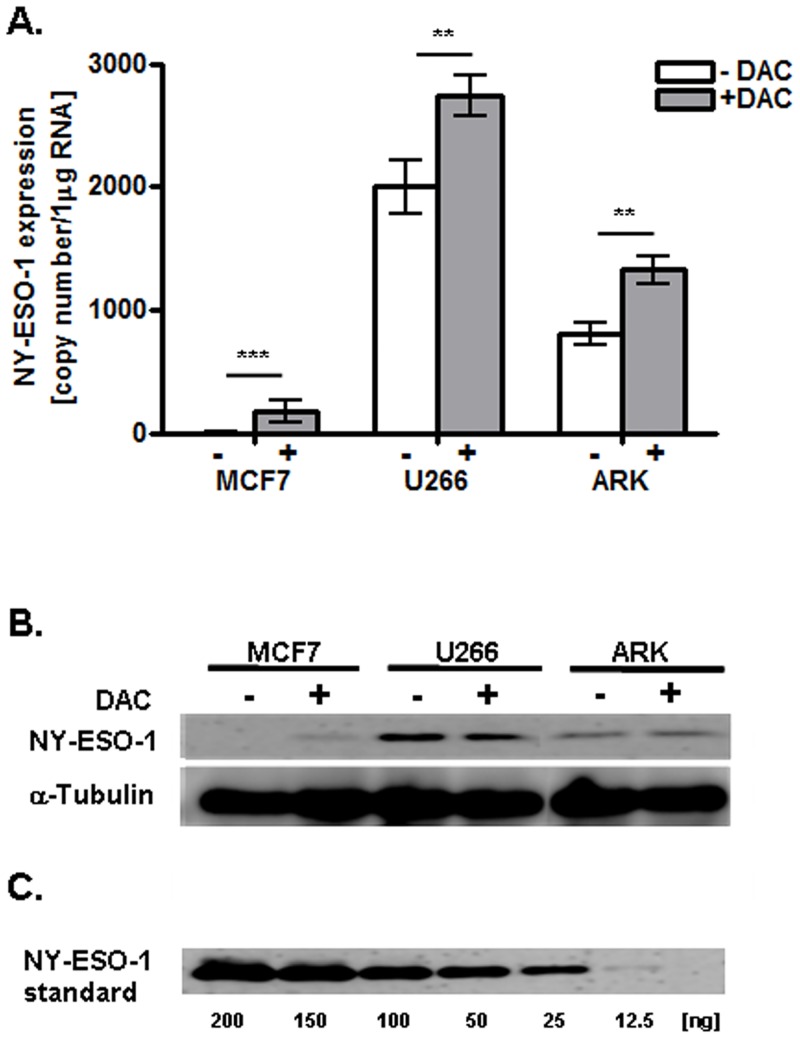
Effects of DAC treatment on NY-ESO-1 mRNA and protein expression in MCF7, U266, and ARK cell lines. A. NY-ESO-1 specific mRNA, quantified as copy numbers/μg RNA using qRT-PCR. All data are representative of three independent experiments performed in triplicate. B. NY-ESO-1 protein expression analyzed by Western blotting (n = 3). The first and second line show total cell lysate of the respective cell line +/- DAC treatment and detection via a NY-ESO-1 specific or α-Tubulin specific (loading control) antibody. The third line shows a Western blot of recombinant NY-ESO-1 protein as control.

The influence of DAC treatment duration (1–3 days) at the antigen surface presentation was further studied (Fig 2C). Antigen expression on MCF7 and U266 cancer cells was already significantly increased after two days of DAC stimulation as compared to untreated cells. However, the strongest statistically significant antigen up-regulation (p <0.0001) was generated after three days of DAC treatment in both breast cancer and multiple myeloma cells. In conclusion, the highest levels of HLA-A2/NY-ESO-1p_157-165_ expression was observed on MCF7 and U266 cancer cells using 10 μM DAC, four times per day, for 3 days. Consequently, subsequent experiments were conducted using this treatment protocol.

### Modulation of NY-ESO-1 mRNA and protein expression in DAC-treated tumor cells

To determine whether the treatment of those tumor cells with DAC would affect the expression of NY-ESO-1 mRNA, cells were treated with 10 μM of DAC four times per day for three days and qRT-PCR was performed (n = 3). MCF7 cells which were initially negative for NY-ESO-1 antigen mRNA by qRT-PCR, showed NY-ESO-1 mRNA induction after DAC treatment (Fig 2A). Moreover, exposure to DAC invariably increased the expression of NY-ESO-1 in both multiple myeloma cell lines (U266, ARK). 18s rRNA (an endogenous control) was equally expressed on all cell lines before and after DAC treatment (data not shown).

To confirm that the induction or increase of NY-ESO-1 expression observed at mRNA level was followed by the production of the respective protein, Western blot for NY-ESO-1 was performed on untreated and DAC-treated tumor cells (n = 3). Treatment induced a *de novo* NY-ESO-1 protein expression in MCF7 cell line (Fig 2B), whereas the protein levels in U266 increased only slightly. NY-ESO-1 protein levels in ARK cells did not demonstrate significant differences before and after DAC-treatment (Fig 2B). Recombinant NY-ESO-1 protein was used as a positive control. On the basis of the NY-ESO-1 standard curve, we assessed the approximate NY-ESO-1 protein levels in treated MCF7 cells with 12.5 ng, U266 cells with 25 ng, and ARK cells with 20 ng, respectively.

### Quantification of cell surface HLA-A2/NY-ESO1p_157-165_ complexes

**Fig 3:**
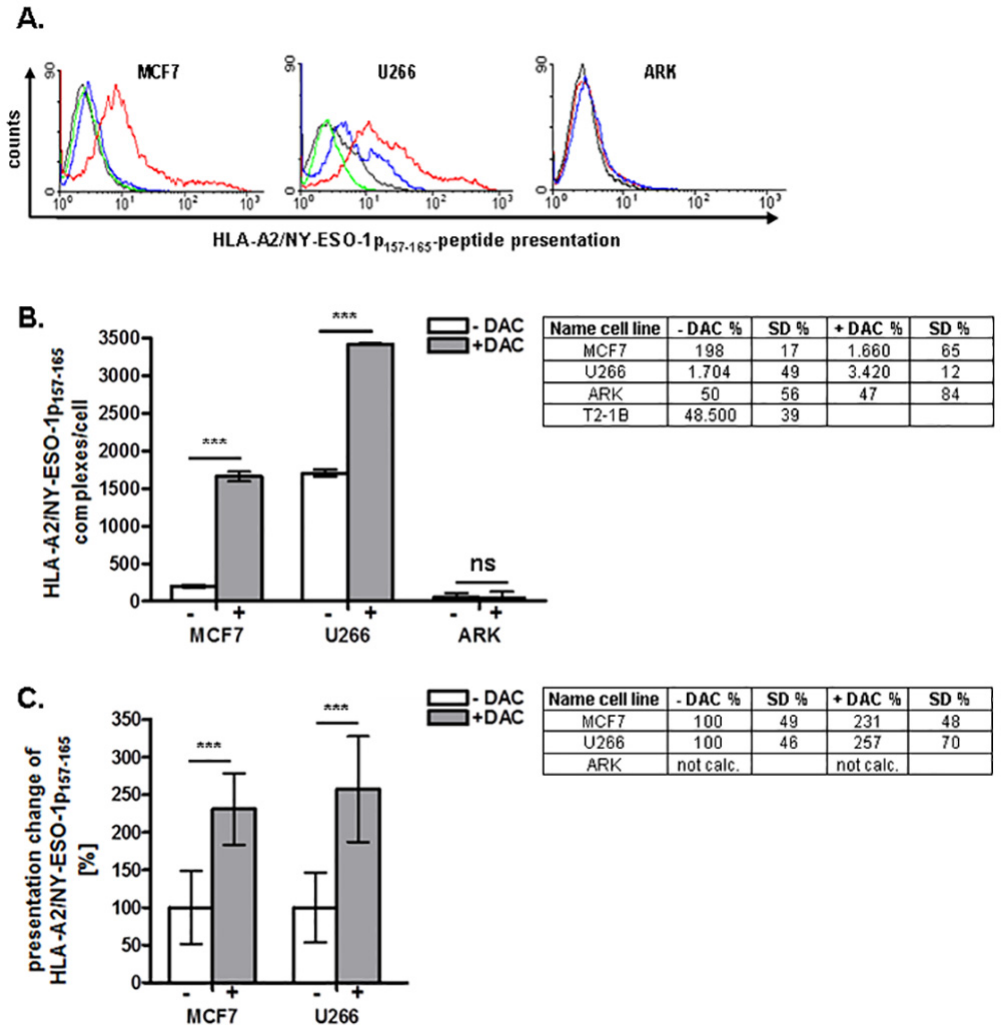
Quantification of HLA-A2/NY-ESO1p_157-165_ complexes at the cell surface of MCF7, U266, and ARK tumor cell lines. A. Flow cytometry histogram after staining of the indicated tumor cell lines with HLA-A2/NY-ESO-1p_157-165_ peptide specific Fab-T1 tetramer (blue/red) and isotype control (black/green). Cells were either untreated (black/blue) or DAC- treated (green/red). B. Quantification of the NY-ESO-1 peptides at the surface of the untreated (white bars) or DAC-treated (grey bars) tumor cell lines. Peptide numbers were calculated as described in materials and methods. C. Relative change in HLA-A2/NY-ESO-1p_157-165_ peptide presentation on the tumor cell lines. Increase of NY-ESO-1p_157-165_ peptide presentation is shown in relation to the total number of HLA-A2 molecules on the tumor cell lines following DAC treatment. ARK cells were used as a negative control. All data are representative of at least five independent experiments performed in triplicate.

HLA-A2/NY-ESO1p_157-165_ protein expression in the untreated and DAC-treated tumor cells MCF7, U266, ARK, and T2-1B (positive control) was assessed by flow cytometry using a specific Fab-T1 tetramer (n = 5) (Fig 3). Histograms (Fig 3A) confirmed an induction of antigen presentation at the surface of MCF7 cells following DAC stimulation, as well as a significantly increased peptide presentation on U266 myeloma cells. No positive fluorescence signal was detected on ARK cells (negative control).

Next, we set out to quantify and compare the number of HLA-A2/NY-ESO1p_157-165_ complexes displayed at the cell surface of untreated and DAC-treated cells. For this purpose, cells were stained with QuantiBRITE PE beads (Fig 3B). To calculate the absolute numbers of PE molecules per cell, the level of fluorescence intensity detected on stained tumor cells was compared with the fluorescence intensities of standard PE calibration beads with known numbers of PE molecules per bead (QuantiBRITE PE beads; BD Biosciences) (S2 Fig). Following DAC treatment, we detected the *de novo* expression of 1,66 x 10^3^ HLA-A2/NY-ESO1p_157-165_ complexes at a MCF7 cell (Fig 3B). The analysis of NY-ESO-1 expression on U266 tumor cells showed a two-fold increase following DAC treatment (from 1,7 x 10^3^ to 3,4 x 10^3^ antigens per cell) (Fig 3B). T2-1B cells were used as a positive control and displayed about 48.5 x 10^3^ peptides per cell as demonstrated in Fig 3B.

### Effect of DAC-treatment on HLA class I molecule density on tumor cells

Due to the HLA-A2-restrictrion of NY-ESO-1 antigen recognition, we analyzed the expression of HLA-A2 molecules at the surface of MCF7, U266, and ARK cells by flow cytometry (n = 5) (n = 5) (S3A Fig). DAC treatment up-regulated the HLA-A2 expression on MCF7 breast cancer cells by 17%, whereas its expression was down-regulated by 22% on U266 myeloma cells when compared to the untreated cells (S3B Fig). The representative quantification of absolute numbers of HLA-A2 molecules per cell were calculated as previously described using QuantiBRITE PE beads (S3B Fig).

### Density of NY-ESO-1 surface complexes in relationship to HLA-A2 molecules on tumor cells

Having assessed both the NY-ESO-1p_157-165_ and HLA-A2 peptide density on tumor cells, we normalized the NY-ESO-1p_157-165_ peptide presentation to HLA-A2 expression (Fig 3C). The antigen expression in the untreated MCF7 and U266 was set to 100%. The relative increase of NY-ESO-1p_157-165_ peptides at the surface of DAC-induced cells, relative to the HLA-A2 expression, was 157% for MCF7 and 131% for U266 cells, respectively. The ARK cell line (HLA-A2-negative) was not used for the calculation (Fig 3C).

### Fluorescence microscopy analysis of NY-ESO-1 expression at the cell surface

**Fig 4:**
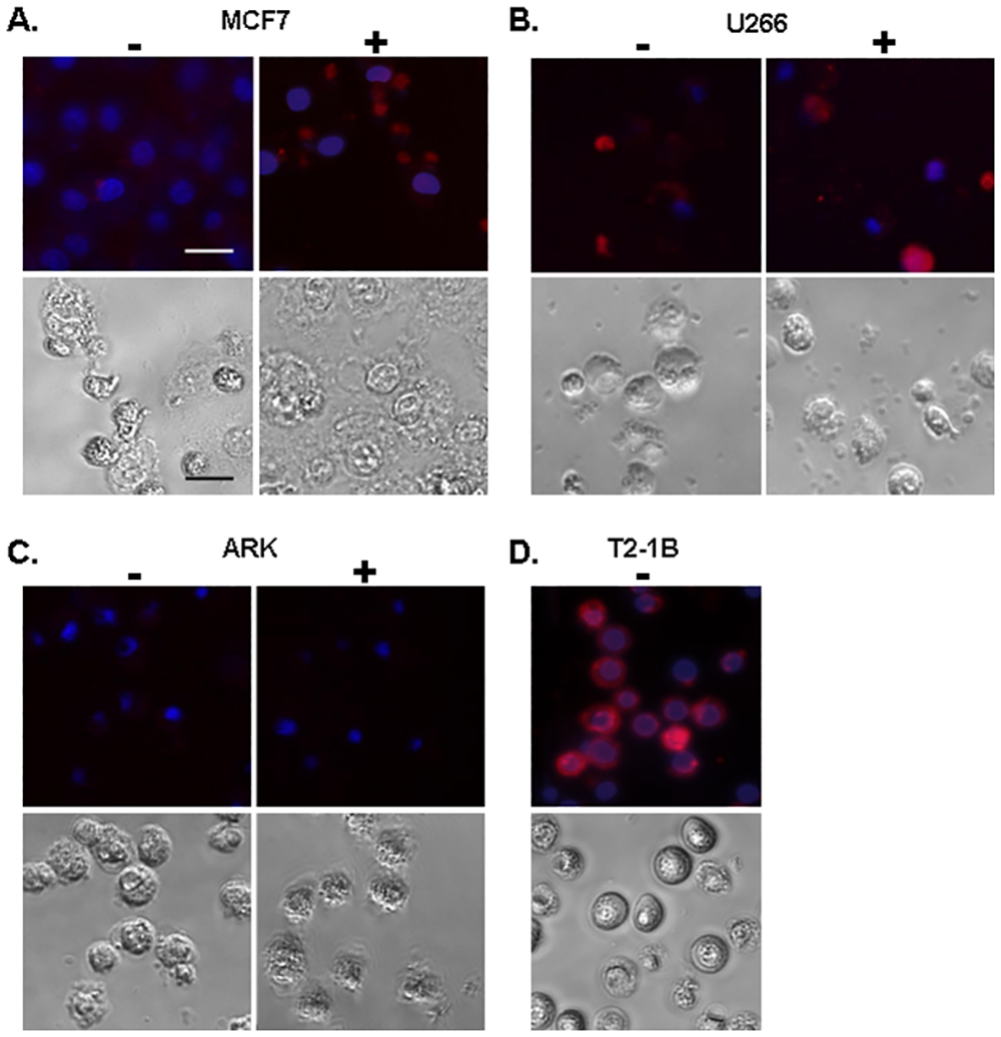
Fluorescence microscopy of HLA-A2/NY-ESO-1p_157-165_ peptide presentation on MCF7-, U266-, ARK- and T2-1B cells. The fluorescence microscopy pictures of the indicated cell lines are shown without (-) or after (+) DAC treatment (n = 5). Cells were stained with the HLA-A2/NY-ESO-1p_157-165_ peptide specific PE-labeled Fab-tetramer (red) and nuclei with DAPI (blue). ARK cells were used as a negative (C) and T2-1B cells as a positive control (D). Scale bar = 25 μm.

In addition to flow cytometry, we determined the NY-ESO-1 antigen presentation on tumor cells using fluorescence microscopy analysis (n = 5) (Fig 4). The complexes were visualized at the surface with HLA-A2/NY-ESO-1_157−165_-specific Fab-tetramer and nuclei were counterstained with DAPI. The NY-ESO-1 peptide expression on MCF7 breast cancer cells in the untreated cells was undetectable and only very weakly stained after DAC-stimulation (Fig 4A). In contrast, U266 cells confirmed NY-ESO-1 positive expression before and after DAC treatment (Fig 4B). ARK and T2-1B lines were used as negative and positive control, respectively (Fig 4C and 4D).

### Enhancement of anti HLA-A2/NY-ESO-1_157–165_ peptide specific effector function of redirected CD8^+^ T cells following DAC treatment

In the previous experiment we have demonstrated that DAC treatment induces or increases the expression HLA-A2/NY-ESO-1_157–165_ peptide presentation on MCF7 and U266 cells. Therefore, we tested whether DAC treatment enhanced the cytolytic recognition by anti-NY-ESO-1 CAR cells as direct consequence of an increased peptide expression (n = 3). We transduced CD8+ T cells with an anti-NY-ESO-1 CAR and anti-CEA CAR as a control. DAC-treated MCF7 cells specifically activated anti-NY-ESO-1 CAR redirected CD8^+^ T cells, whereas almost no lysis was observed with untreated MCF7 cells. We observed a low cytotoxic effect of control anti-CAR redirected T cells with untreated MCF7 cells at high levels of effector cells (2:1 E:T ratio) [33,34]. This effect was not detected using lower E:T ratios such as 1:1 and 0.5:1.

**Fig 5:**
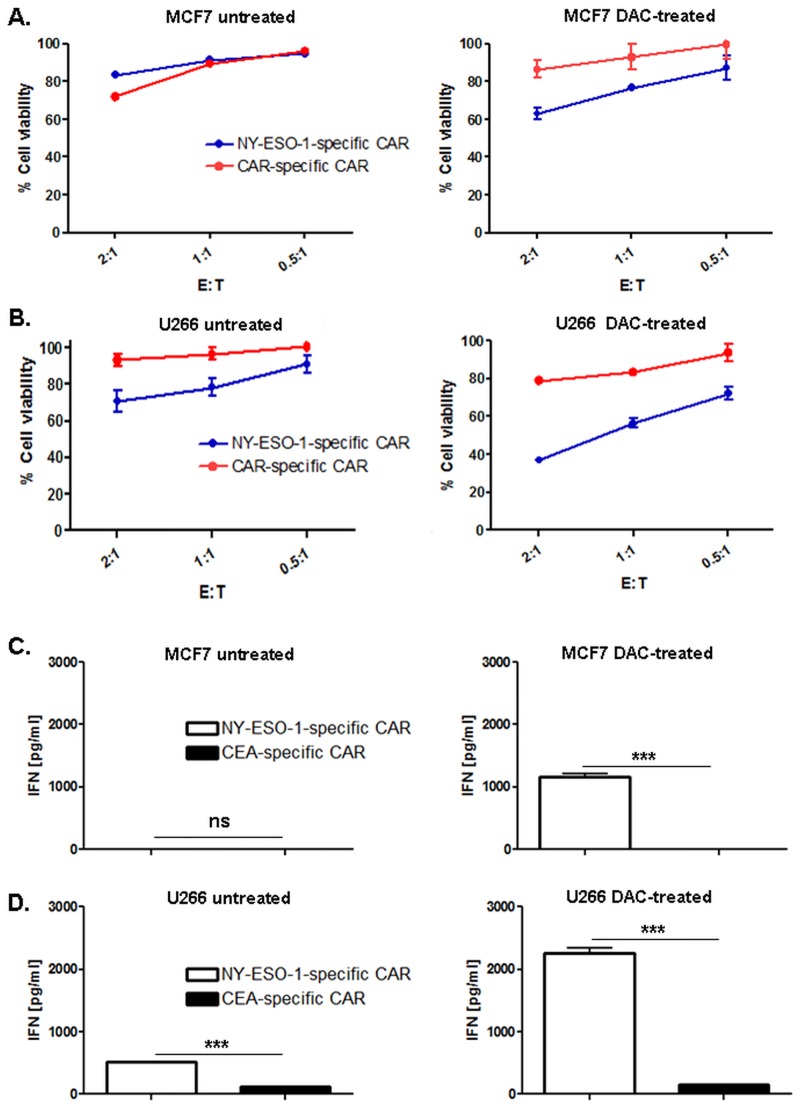
Increased specific lysis of MCF7 and U266 tumor cells by CAR redirected CD8^+^ T cells after DAC treatment. Retrovirally transduced NY-ESO-1-specific and CEA-specific CAR redirected CD8^+^ T cells were cocultivated with U266 or MCF7 cells. NY-ESO-1 expression after DAC treatment statistically significantly enhanced the antigen specific killing of anti-NY-ESO-1 CAR redirected T cells in U266 (A), whereas the increased lysis of MCF7 was only detectable after DAC treatment (B). Antigen specific activation of anti-NY-ESO-1 CAR redirected CD8^+^ T cells was determined by IFN-gamma (C and D). All data are representative of three independent experiments performed in triplicate.

The multiple myeloma cell line U266 that constitutively expresses NY-ESO-1 protein was lysed even in the absence of DAC treatment. However, DAC treatment enhanced this lysis significantly, especially using at the E:T ratio 2:1 and 1:1 (p<0.001), respectively. No specific lysis was observed with control CAR redirected T cells with U266 cells or HLA-A2 negative ARK cells (Fig 5A and 5B; data not shown, respectively). T2-1B cells were used as a positive control (S5A Fig).

In addition, we measured the T cell activation using IFN-gamma secretion in the culture supernatant of both DAC-treated and untreated samples. Specific IFN-gamma secretion was observed with DAC-treated MCF7 cells and untreated/treated U266 cells. However, the highest IFN-gamma secretion was detected in U266 cells following DAC-treatment (Fig 5D) and T2-1B cells (S5B Fig). These results indicate that increasing peptide density at the cell surface after DAC-treatment enhanced the antigen specific function of redirected T cells.

## Discussion

In this study, we visualized the HLA-A2-restricted NY-ESO-1_157–165_ peptide expression at the cell surface of tumor cells using a high affinity soluble TCR construct. We demonstrated that DAC treatment clearly induced or up-regulated NY-ESO-1 expression in a dose- and time-dependent manner on mRNA, protein, and peptide presentation level. In addition, the efficient recognition of DAC-treated tumor cells by specific CAR T cells confirms that *de novo* synthesized NY-ESO-1 antigen is functionally active as judged by peptide presentation. Finally, we quantified the total number of NY-ESO-1_157–165_ peptides presented on tumor cells lines relatively to the amount of HLA molecules showing the physiological surface densities of this antigen.

The highest NY-ESO-1 antigen up-regulation in breast and multiple myeloma cancer cells was achieved in our study with 10 μM DAC, 4 times daily for 4 consecutive days. In comparison, in another study with orthotopic human glioma cancer cells, only 2 days of treatment with 0.1–10 μM DAC were used to induce NY-ESO-1 expression [12], whereas neuroblastoma cells required 5 days exposure to DAC [35]. Ovarian cancer cells needed even longer with a 7-day DAC treatment at 1–3 μM for up-regulation of distinct CTAs [36]. Since DAC acts only on proliferating cells, the first effects of treatment occur routinely after at least two doubling times [37]. This explains different DAC treatment requirements regarding various cancer cell lines and the associated demethylation rate of a newly synthesized DNA strand [36]. However, it is also evident that there are differences in the response to DAC treatment, as in some cancer cell lines used in this study there was no significant effect of the treatment on the NY-ESO-1 presentation [38]. We believe that it might be due to the high heterogeneity among different cancer including melanoma [38,39], myeloma [40], and breast cancer cells [41].

Using the optimized time- and dose-dependent DAC protocol, we observed the *de novo* induction or up-regulation of NY-ESO-1 expression on mRNA and protein level. These results are consistent with other *in vitro* studies using distinct cancer cell lines [12,16,36]. Interestingly, the increase of NY-ESO-1 peptide presentation did not directly correlate with gene expression following DAC treatment. Whereas the increase of NY-ESO-1 peptide presentation on breast cancer cells was lower than the mRNA expression, the peptide density on multiple myeloma cells was higher in comparison to the corresponding increase in gene expression. These findings imply that there are other factors regulating the translation and posttranslational modifications of NY-ESO-1 mRNA leading to a non-linear relationship between mRNA and peptide presentation levels. Other possible explanation could be that multiple myeloma U266 cells may show an increased rate of proteasomal degradation and antigen presentation.

Since NY-ESO-1_157–165_ antigen is presented in a HLA-A2 restricted manner, we set ought to quantify the expression of both molecules after exposure to DAC treatment. Interestingly, we determined that NY-ESO-1_157–165_ antigen presentation levels were largely unaffected by induced up-regulation of HLA-A2 levels, indicating that NY-ESO-1 processing and presentation, and not HLA levels, is a limiting factor regarding the expression of this antigen. A similar finding was reported in previous studies in the context of NY-ESO-1 expression in melanoma cells [16,42]. This aspect is of great advantage for anticancer immunotherapies as tumor cells usually down-regulate the expression of HLA class I levels to evade immune surveillance [43,44,45].

The efficient recognition of DAC-treated breast cancer cells by the HLA-A2 restricted NY-ESO-1 specific CAR T cells confirmed in our study that the *de novo* synthesized NY-ESO-1 antigen is functionally processed and presented. In addition, DAC-treated multiple myeloma cells elicited a robust immunogenic stimulus as demonstrated by both ELISA and cytotoxicity assays. These results are consistent with previous observations reporting an enhanced lysis of DAC-treated human ovarian [36] or glioma [12] cells.

In conclusion, the susceptibility of DAC-treated tumor cell lines to antigen-specific T cell recognition strongly identifies DAC as a potential pharmacological agent for future clinical trials in combination with specific immunotherapies.

## Supporting Information

S1 Fig Susceptibility of different cancer cell lines to DAC treatment. A. The expression status of NY-ESO-1_157–165_ and HLA-A*0201 (HLA-A2) on different human cell lines: melanoma (SK-MEL-23, SK-MEL-37), breast cancer (ALAB, MCF7, 734B), and multiple myeloma (ARK, U266). B. Flow cytometry analysis of HLA-A2/NY-ESO-1p_157-165_ expression on indicated cancer cell lines. Cells were treated for 2 days with 10 μM DAC (+), 2x per day or without DAC (-) and analyzed by flow cytometry with an isotype (white and white dashed) or HLA-A2 / NY-ESO-1_157−165_ specific (grey and dark-grey) Fab-T1 tetramer. Mean ± SD; n = 5 independent experiments (n = 3 per condition).
(TIF)

S2 Fig Surface antigen quantification by standard QuantiBrite PE beads. A. Flow cytometric histogram representation of the fluorescent beads (left) and the calculation of fluorescence molecules corresponding to the mean fluorescence intensity (MFI) of each peak (right). B. Standard curve representing the number of fluorescent molecules versus MFI.
(TIF)

S3 Fig Quantification of HLA-A2 molecules at the cell surface of MCF7, U266, and ARK cells. A. Flow cytometric analysis of HLA-A2-expression shown as a histogram representation. All diagrams show curves of untreated (black and blue) and DAC-treated cells (green and red), stained with an isotype- (black and green) or HLA-A2 / NY-ESO-1_157−165_ specific (blue and red) Fab-T1 tetramer. Mean ± SD; n = 5 independent experiments (n = 3 per condition).
(TIF)

S4 Fig Surface expression of chimeric antigen receptor on human CD8^+^ T cells confirmed by FACS analysis. Transduced CD8+ T cells were simultaneously incubated with FITC-conjugated anti-CD8 mAb and PE- conjugated anti-human IgG.
(TIF)

S5 Fig Specific lysis of T2-1B cells by CAR redirected CD8^+^ T cells. A. Retrovirally transduced NY-ESO-1-specific CAR redirected CD8^+^ T cells showed specific killing after coculture with T2-1B cells. B. IFN-gamma secretion was used to determine the antigen specific activation of NY-ESO-1-specific CAR redirected CD8^+^ T cells. Mean ± SD; all data are representative of three independent experiments performed in triplicate.
(TIF)
