# Supplementary figures and images for: Treatment with 5-Aza-2'-Deoxycytidine Induces Expression of NY-ESO-1 and Facilitates Cytotoxic T Lymphocyte-Mediated Tumor Cell Killing
Source: PLoS One. 2015 Oct 8;10(10):e0139221. doi: 10.1371/journal.pone.0139221 (PMC4598131; doi:10.1371/journal.pone.0139221)

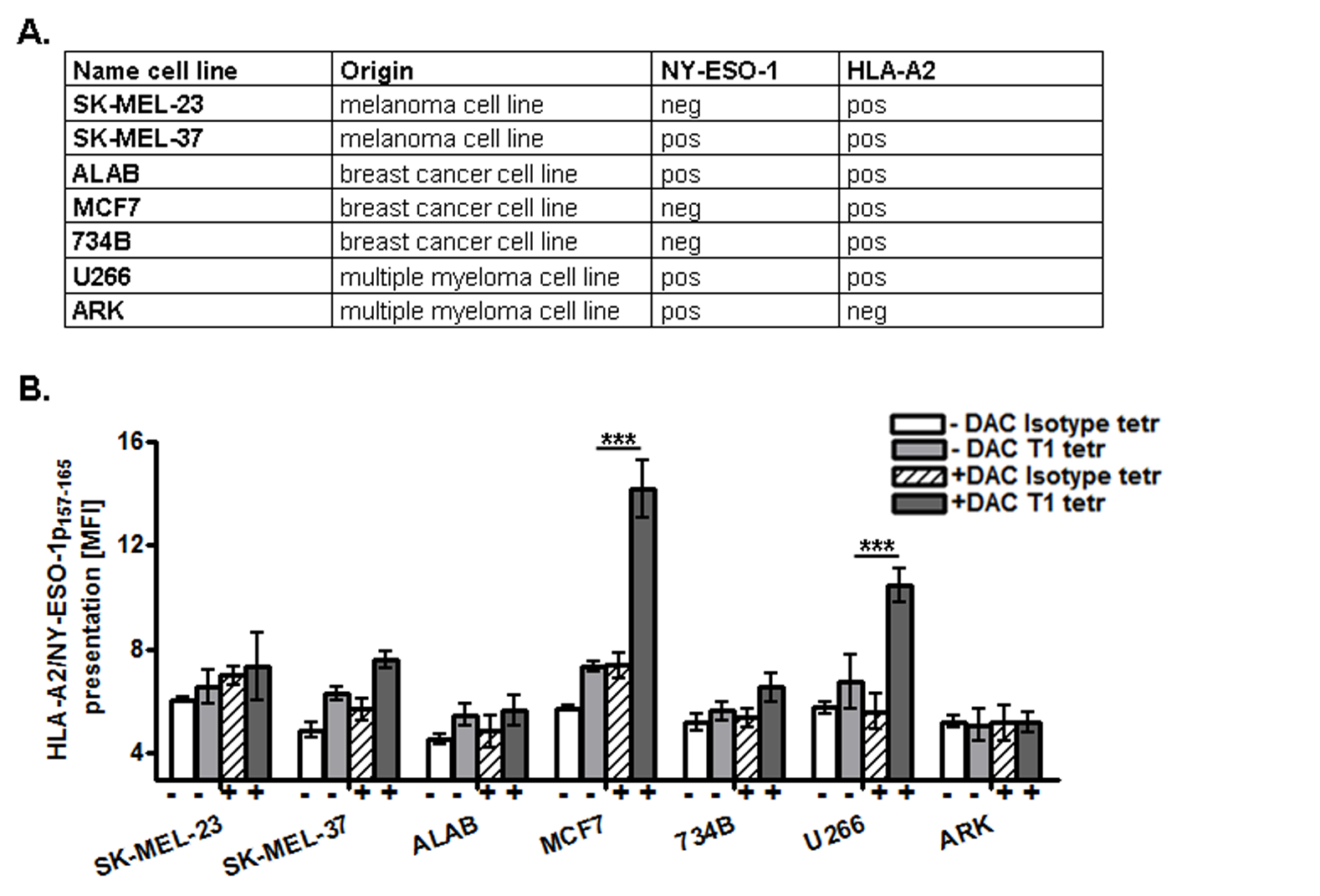

Supplement: S1 Fig — A. The expression status of NY-ESO-1157–165 and HLA-A*0201 (HLA-A2) on different human cell lines: melanoma (SK-MEL-23, SK-MEL-37), breast cancer (ALAB, MCF7, 734B), and multiple myeloma (ARK, U266). B. Flow cytometry analysis of HLA-A2/NY-ESO-1p157-165 expression on indicated cancer cell lines. Cells were treated for 2 days with 10 μM DAC (+), 2x per day or without DAC (-) and analyzed by flow cytometry with an isotype (white and white dashed) or HLA-A2 / NY-ESO-1157−165 specific (grey and dark-grey) Fab-T1 tetramer. Mean ± SD; n = 5 independent experiments (n = 3 per condition). (TIF) [file pone.0139221.s001.tif]

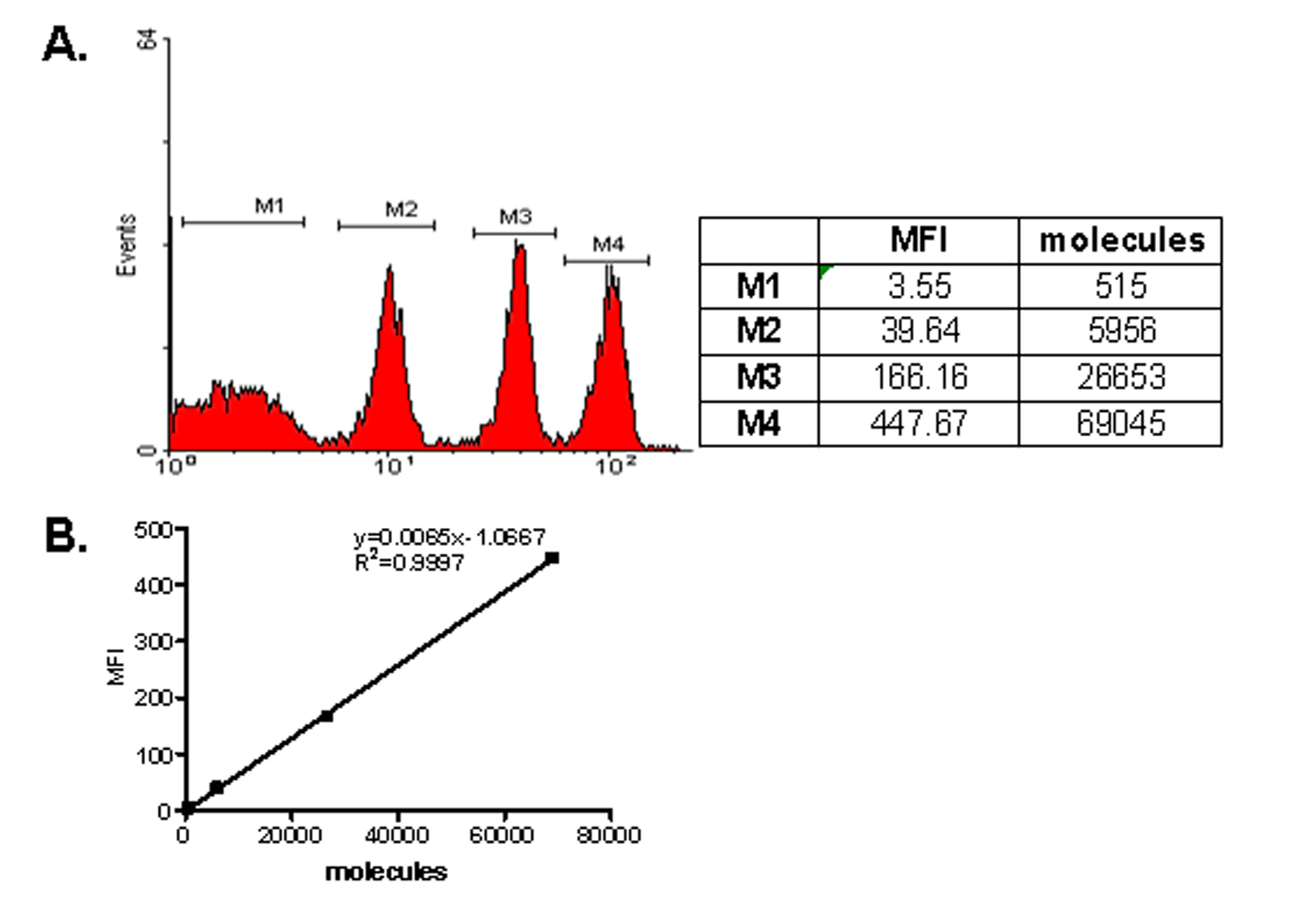

Supplement: S2 Fig — A. Flow cytometric histogram representation of the fluorescent beads (left) and the calculation of fluorescence molecules corresponding to the mean fluorescence intensity (MFI) of each peak (right). B. Standard curve representing the number of fluorescent molecules versus MFI. (TIF) [file pone.0139221.s002.tif]

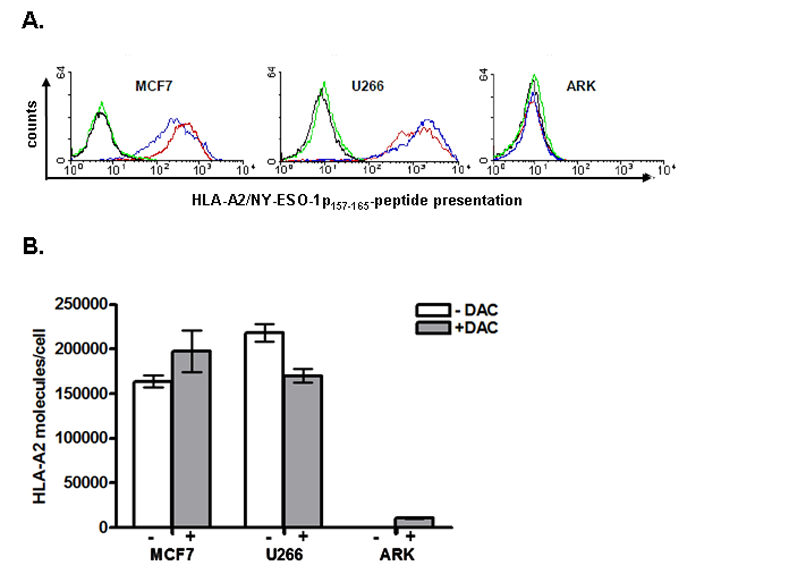

Supplement: S3 Fig — A. Flow cytometric analysis of HLA-A2-expression shown as a histogram representation. All diagrams show curves of untreated (black and blue) and DAC-treated cells (green and red), stained with an isotype- (black and green) or HLA-A2 / NY-ESO-1157−165 specific (blue and red) Fab-T1 tetramer. Mean ± SD; n = 5 independent experiments (n = 3 per condition). (TIF) [file pone.0139221.s003.tif]

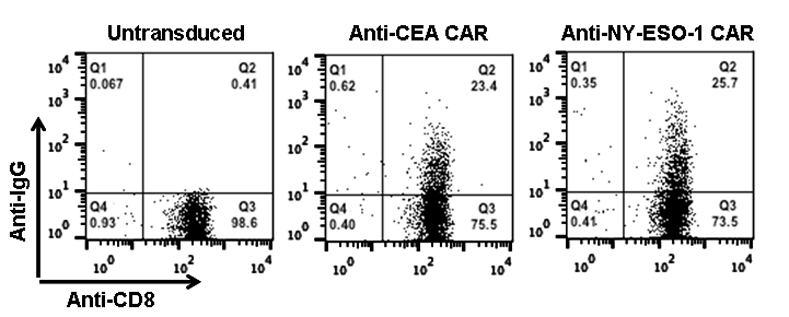

Supplement: S4 Fig — Transduced CD8+ T cells were simultaneously incubated with FITC-conjugated anti-CD8 mAb and PE- conjugated anti-human IgG. (TIF) [file pone.0139221.s004.tif]

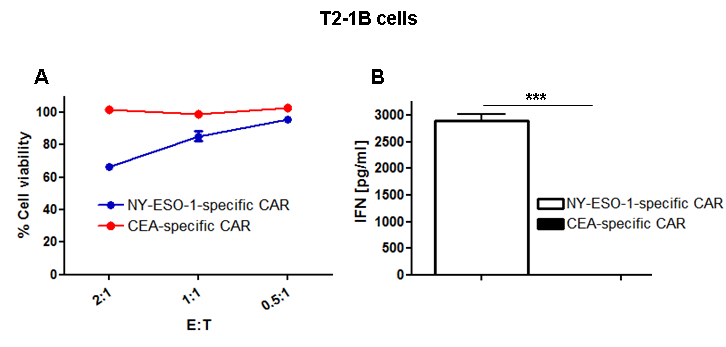

Supplement: S5 Fig — A. Retrovirally transduced NY-ESO-1-specific CAR redirected CD8+ T cells showed specific killing after coculture with T2-1B cells. B. IFN-gamma secretion was used to determine the antigen specific activation of NY-ESO-1-specific CAR redirected CD8+ T cells. Mean ± SD; all data are representative of three independent experiments performed in triplicate. (TIF) [file pone.0139221.s005.tif]
